# A multicenter analysis to identify the risk factors for stroke recurrence and mortality within 1 year

**DOI:** 10.3389/fneur.2025.1478175

**Published:** 2025-02-05

**Authors:** Eman A. Alraddadi, Hashim F. Alotaibi, Yasser Alatawi, Ahmed Aljabri, Ahmed A. Alghamdi, Fahad Alturki, Faisal F. Alamri

**Affiliations:** ^1^Department of Basic Sciences, College of Science and Health Professions, King Saud Bin Abdulaziz University for Health Sciences, Jeddah, Saudi Arabia; ^2^King Abdullah International Medical Research Center, Jeddah, Saudi Arabia; ^3^College of Medicine, King Saud Bin Abdulaziz University for Health Sciences, Riyadh, Saudi Arabia; ^4^Department of Pharmacy Practice, Faculty of Pharmacy, University of Tabuk, Tabuk, Saudi Arabia; ^5^Department of Pharmacy Practice, Faculty of Pharmacy, King Abdulaziz University, Jeddah, Saudi Arabia

**Keywords:** stroke recurrence, mortality, ischemic stroke, risk factors, stroke complication

## Abstract

**Background:**

Stroke recurrence is a serious and prevalent complication of ischemic stroke that warrants additional investigation.

**Methods:**

A hospital-based retrospective observational study included acute-subacute ischemic stroke adult patients. The primary aim was to determine the risk factors associated with recurrent stroke within 365 days. Additionally, a combined outcome consisting of any stroke recurrence or all-cause mortality within 365 days was considered secondary outcome. Univariate and multivariable Cox proportional-hazards models were used to examine the association of risk factors with stroke recurrence and composite death/stroke recurrence.

**Results:**

Of 1,244 patients, 112 (9%) experienced stroke recurrence. The multivariable analysis identified risk factors for stroke recurrence including history of previous stroke (HR = 3.65, 95% CI:2.28–5.99, *p* = 0.0001), tissue plasminogen activator (tPA) treatment (HR = 2.84, 95% CI:1.57–4.86, *p* = 0.0003), seizure (HR = 1.96, 95% CI:1.14–3.22, *p* = 0.0105), and depression (HR = 2.26, 95% CI:1.33–3.69, *p* = 0.0016). Only previous stroke history (HR = 2.37, 95% CI:1.74–3.26, *p* = 0.0001) remained significantly associated with the combined outcome of stroke recurrence/death. Additional risk factors for the composite outcome included older age of patients (HR = 1.02, 95% CI:1.01–1.03, *p* = 0.0009), admission to the intensive care unit (ICU) (HR = 3.70, 95% CI:2.63–5.22, *p* = 0.0105), pneumonia (HR = 1.47, 95% CI:1.05–2.05, *p* = 0.0249), and brain edema (HR = 2.36, 95% CI:1.58–3.46, *p* = 0.0001).

**Conclusion:**

Key findings include a stroke recurrence rate of 9.96% and a combined death/stroke recurrence rate of 21.83% within 365 days. Multivariable analysis confirmed that history of stroke, receiving tPA, experiencing seizures, and depression were significantly associated with stroke recurrence. Implementing additional preventive measures for individuals in these high-risk categories is essential. Further studies are needed to validate our findings.

## 1 Introduction

Stroke stands as the global second-leading cause of death and the third-leading cause of combined mortality and disability (1). It represents a significant health burden, with an increased global incidence rate of 70% over the last three decades (1). Ischemic stroke is the most prevalent type, accounting for 87% of all stroke cases. Recurrent stroke presents an ominous complication for patients who have experienced an initial ischemic stroke (2). Compared to initial stroke, recurrence stroke is more difficult to treat and carries higher mortality rates. Despite advancements in clinical care, the risk of recurrence remains alarmingly high, with nearly 40% of patients experiencing a recurrent stroke within 5 years (3). Therefore, prevention is of considerable importance to both individual and public health. Early identification of recurrent stroke and optimal management are essential to improve patient care and quality of life.

A thorough understanding of the risk factors for stroke recurrence is vital for enhancing secondary prevention strategies. On a global scale, hypertension emerges as the most dominant risk factor, followed by hyperlipidemia, ischemic heart disease, atrial fibrillation (AF), and diabetes mellitus (DM) (3, 4). In addition, other studies have identified hypercholesterolemia and obesity as significant risk factors for recurrent stroke (5, 6). Locally, one prospective study held in one region of Saudi Arabia identified hypertension and DM as substantial risk factors for recurrent stroke, with both conditions coexisting in a high proportion of recurrent stroke patients. Ischemic heart disease was featured prominently in almost half of the enrolled subjects, as well as smoking and AF (7).

Despite the existing evidence related to some of the risk factors associated with the incidence of recurrent stroke, many potential contributors have not been extensively investigated. Thus, this study aimed to determine the incidence and contributing risk factors of stroke recurrence among patients with acute and subacute ischemic stroke in two tertiary hospitals.

## 2 Patients and methods

### 2.1 Study design

**Figure 1:**
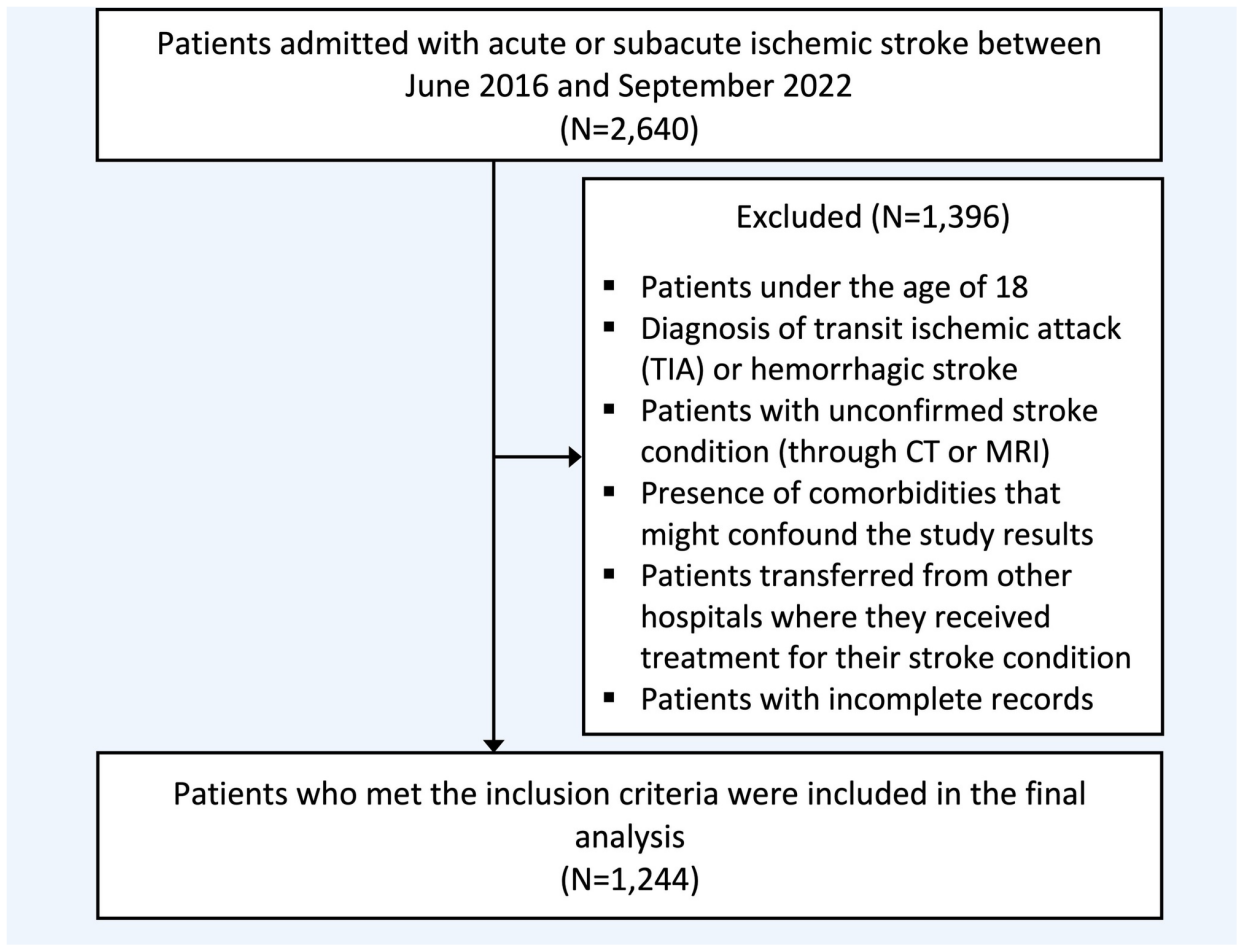
Flowchart of study population selection. CT, Computed tomography; MRI, Magnetic resonance imaging.

A hospital-based retrospective observational study was carried out on individuals with acute and subacute ischemic stroke treated at two tertiary hospitals in different cities between June 2016 and September 2022. Electronic medical records (BESTCare® 2.0) were used to screen eligible patients based on the inclusion and exclusion criteria, Figure 1. Data was extracted using a secured data collection sheet in Excel.

### 2.2 Study participants

Inclusion criteria comprised patients who are 18 years or older and arrived at the emergency room within 7 days of stroke symptoms or were admitted at the incident time with a confirmed ischemic stroke diagnosis using either a magnetic resonance imaging (MRI) or a computed tomography (CT) scan. Exclusion criteria comprised patients with a diagnosis of transient ischemic attack (TIA) or hemorrhagic stroke, the presence or history of significant comorbidities that could confound the study results (intracranial pathology, tumors, or traumatic brain injury), those transferred from other hospitals, and individuals with missing or incomplete medical or laboratory records, Figure 1.

### 2.3 Outcomes

Patients were followed up retrospectively for up to 1 year. The primary outcome was the incidence of stroke recurrence within 365 days in patients with ischemic stroke. The definition of stroke recurrence is ischemic or hemorrhagic stroke qualifying for the following conditions: A new neurological deficit or deterioration of the previous deficit not considered to be due to toxic effects of drug therapy, hemorrhagic transformation, or concomitant acute brain injury occurring within 7 days of the index stroke (8, 9). Furthermore, we also considered a combined secondary outcome comprising death or stroke recurrence within 365 days following the initial stroke onset. Death was defined as mortality from any cause (10).

### 2.4 Data collection and potential confounders

The collected data included demographic variables such as age and gender, date of arrival, and date of stroke. Potential modifiable risk factors included the National Institutes of Health Stroke Scale (NIHSS) score, previous history of stroke, intensive care unit (ICU) admission, and comorbidities, including hypertension, DM, dyslipidemia, AF, dementia, and depression (11). In addition, data regarding treatment intervention, including mechanical thrombectomy and tissue-type plasminogen activator (tPA), were also collected. Other potential confounders included post-stroke complications such as seizure, pneumonia, cerebral edema, deep vein thrombosis-pulmonary embolism (DVT-PE), and impaired consciousness. The stroke subtype was determined according to the Trial of Org 10172 in Acute Stroke Treatment (TOAST) classification. The TOAST classification denotes five subtypes of ischemic stroke: (1) large-artery atherosclerosis, (2) cardioembolism, (3) small-vessel occlusion, (4) stroke of other determined etiology, and (5) stroke of undetermined etiology (9).

### 2.5 Statistical analysis

Normally distributed continuous variables were presented as mean ± standard deviation, while non-normally distributed variables were expressed as median and interquartile range (IQR). Gray’s Test for Equality of Cumulative Incidence Functions was employed to assess the equality of cumulative risks. Cox proportional-hazards models were used to analyze univariate associations of risk factors with stroke recurrence and death/stroke recurrence. Furthermore, multivariable Cox proportional-hazards models with the backward elimination method were used to examine the association of risk factors with stroke recurrence and death/stroke recurrence. Patients were monitored until the earliest occurrence of stroke recurrence, death, or the censoring date, defined as either 1 year from the index date (stroke onset) or September 1, 2023, whichever came first. Censoring for stroke recurrence occurred if the cause of death was unspecified or attributed to a cause other than stroke. All statistical analyses were performed using SAS software version 9.4.

## 3 Results

### 3.1 Baseline and clinical characteristics of patients included in the study

A total of 2,640 patients underwent screening, with 1,244 included in the final analysis, Table 1. The mean age was 66 years, and 38.26% were female. Patients with a history of more than one stroke at baseline accounted for 39.31% of the sample. The median NIHSS score, reflecting initial clinical severity, was 6 (IQR: 3–10). The rate of individuals receiving tPA and mechanical thrombectomy was 9.44 and 5.08%, respectively. ICU admission during hospitalization was documented in 30.20% of the sample. The reported comorbidities included hypertension (*n* = 1,016, 81.80%), DM (*n* = 917, 73.83%), dyslipidemia (*n* = 559, 45.01%), AF (*n* = 132, 10.63%), dementia (*n* = 76, 6.12%), and depression (*n* = 160, 12.92%). Reported post-stroke complications were seizure (*n* = 168, 13.57%), pneumonia (*n* = 279, 22.50%), cerebral edema (*n* = 103, 8.32%), DVT-PE (*n* = 101, 8.15%), and impaired consciousness (*n* = 362, 29.17%).

**Table 1:** Included patients’ basic and clinical characteristics.

Characteristics	
Stroke Recurrence, *n* (%)	112 (9.00)
Age, mean ± SD	66 ± 13
Male, *n* (%)	768 (61.74)
Stroke subtype, *n* (%)	
1. (large-artery atherosclerosis)	564 (51.18)
2. (cardioembolism)	276 (25.05)
3. (small-vessel occlusion)	70 (6.35)
4. (other determined etiology)	28 (2.54)
5. (undetermined etiology)	164 (14.88)
Unknown/missing	142
Prior stroke, *n* (%)	480 (39.31)
Initial NIHSS, Median (IQR)	6 (3–10)
tPA therapy, *n* (%)	117 (9.44)
Mechanical thrombectomy, *n* (%)	36 (5.08)
ICU admission, *n* (%)	373 (30.20)
Post-stroke complication	
Seizure, *n* (%)	168 (13.57)
Pneumonia, *n* (%)	279 (22.50)
Cerebral edema, *n* (%)	103 (8.32)
DVT-PE, *n* (%)	101 (8.15)
Impaired consciousness, *n* (%)	362 (29.17)
Comorbidities	
Hypertension, *n* (%)	1,016 (81.80)
Diabetes mellitus, *n* (%)	917 (73.83)
Dyslipidemia, *n* (%)	559 (45.01)
Atrial fibrillation, *n* (%)	132 (10.63)
Dementia, *n* (%)	76 (6.12)
Depression, *n* (%)	160 (12.92)

SD, standard deviation; NIHSS, National Institues of Health Stroke Scale; IQR, Interquartile range; tPA, tissue plasminogen activator; ICU, intensive care unit; DVT-PE, deep vein thrombosis-pulmonary embolism.

### 3.2 Cumulative risk of developing stroke recurrence or death/stroke recurrence

The incidence rate of stroke recurrence in the first 365 days after stroke was 9.96% (95% CI: 8.29–11.80). Incidence rates at specific intervals were as follows: 2.55% (95% CI: 1.76–3.57) at 30 days, 5.29% (95% CI: 4.10–6.68) at 90 days, and 6.91% (95% CI: 5.53–8.48) at 180 days. Previous history of stroke was significantly associated with an increased risk of recurrence (*p* = 0.0001). Recurrence rates for patients with a prior stroke history were notably higher compared to those without, at 30 days (4.24% vs. 1.56%), 90 days (8.48% vs. 3.45%), 180 days (11.41% vs. 4.19%), and 365 days (17.21% vs. 5.70%), Table 2.

**Table 2:** Cumulative risks of stroke recurrence and stroke recurrence or death.

	Stroke Recurrence, % (95% CI)				*p*	Death/Stroke Recurrence, % (95% CI)				*p*
	30 Days	90 Days	180 Days	365 Days		30 Days	90 Days	180 Days	365 Days	
At risk, *n*	1,130	1,062	1,045	959		1,130	1,062	1,045	959	
Incidence, *n*	30	61	79	112		100	167	205	268	
All patients	2.55(1.76–3.57)	5.29(4.10–6.68)	6.91(5.53–8.48)	9.96(8.29–11.80)		8.13(6.69–9.75)	13.60(11.75–15.58)	16.69(14.67–18.84)	21.83(19.56–24.18)	
History of stroke										
Yes	4.24(2.64–6.40)	8.48(6.10–11.34)	11.41(8.61–14.63)	17.21(13.75–21.00)	**0.0001**	11.04(8.41–14.07)	18.91(15.51–22.58)	24.02(20.26–27.97)	31.68(27.52–35.92)	**0.001**
No	1.56(0.83–2.69)	3.45(2.27–5.00)	4.19(2.87–5.87)	5.70(4.13–7.62)		6.52(4.89–8.46)	10.60(8.51–12.96)	12.37(10.11–14.87)	16.04(13.49–18.79)	
Stroke subtype										
1 (large-artery atherosclerosis)	2.85(1.67–4.55)	6.20(4.33–8.50)	8.21(6.04–10.80)	11.75(9.11–14.74)	0.3550	8.82(6.64–11.36)	15.50(12.62–18.64)	18.75(15.62–22.10)	24.52(21.02–28.18)	**0.0001**
2 (cardioembolism)	1.85(0.69–4.04)	3.71(1.90–6.48)	4.46(2.4–7.42)	7.17(4.47–10.70)		2.93 (1.38–5.46)	5.86(3.49–9.09)	8.06(4.34–10.39)	12.82(9.18–17.09)	
3 (small-vessel occlusion)	1.45(0.12–6.96)	3.12(0.57–9.73)	4.79 (1.24–12.19)	9.98(4.01–19.19)		12.86(6.28–21.87)	17.14(9.37–26.88)	21.14(12.67–31.71)	27.14(17.30–37.94)	
4 (other determined etiology)	3.85(0.26–16.77)	3.85(0.26–16.77)	3.85(0.26–16.77)	12.04(2.91–28.17)		10.71(2.63–25.38)	14.29(4.37–29.83)	14.29(4.37–29.83)	25.00(10.82–42.14)	
5 (undetermined etiology)	2.70(0.89–6.32)	7.15(3.64–12.24)	8.71(4.73–14.18)	11.95(7.12–18.13)		15.41(10.34–21.42)	24.06(17.77–30.88)	27.76(21.08–34.82)	33.32(26.16–40.62)	

CI, confidence interval. *p*-values < 0.05 written in bold indicating statistical significant difference.

The incidence rate of death or stroke recurrence within the first 365 days after stroke was 21.83% (95% CI: 19.56–24.18). Cumulative incidence rates at specific time intervals were as follows: 8.13% (95% CI: 6.69–9.75) at 30 days, 13.60% (95% CI: 11.75–15.58) at 90 days, and 16.69% (95% CI: 14.67–18.84) at 180 days. Patients with a history of stroke experienced significantly higher rates of death or stroke recurrence compared to those without a prior history. Incidence rates were markedly higher across all periods: 11.04% vs. 6.52% at 30 days, 18.91% vs. 10.60% at 90 days, 24.02% vs. 12.37% at 180 days, and 31.68% vs. 16.04% at 365 days. Furthermore, variations in stroke subtypes significantly influenced the cumulative incidence of death or stroke recurrence over time.

### 3.3 Risk factors associated with stroke recurrence

Univariate analysis (Table 3) identified several risk factors significantly associated with stroke recurrence. These included age (HR = 1.03, 95% CI: 1.01–1.03, *p* < 0.0001), history of stroke (HR = 3.17, 95% CI: 2.16–4.73, *p* < 0.0001), stroke subtype two vs. one as a reference (HR = 0.59, 95% CI: 0.35–0.98, *p* = 0.0477), tPA treatment (HR = 2.07, 95% CI: 1.23–3.31, *p* = 0.0038), ICU admission (HR = 1.89, 95% CI: 1.28–2.77, *p* = 0.0012), seizures (HR = 2.52, 95% CI: 1.63–3.78, *p* < 0.0001), pneumonia (HR = 1.58, 95% CI:1.03–2.37, *p* = 0.0305), brain edema (HR = 1.93, 95% CI:1.01–3.38, *p* = 0.0311), DVT-PE (HR = 1.86, 95% CI:1.01–3.14, *p* = 0.0306), hypertension (HR = 1.95, 95% CI:1.12–3.73, *p* = 0.0294), dyslipidemia (HR = 1.70, 95% CI:1.17–2.50, *p* = 0.0057), and depression (HR = 2.56, 95% CI:1.66–3.85, *p* < 0.0001). Upon multivariable analysis (Table 4), risk of stroke recurrence remained significantly associated with a prior history of stroke (HR = 3.65, 95% CI:2.28–5.99, *p* = 0.0001), tPA (HR = 2.84, 95% CI:1.57–4.86, *p* = 0.0003), seizure (HR = 1.96, 95% CI:1.14–3.22, *p* = 0.0105), and depression (HR = 2.26, 95% CI:1.33–3.69, *p* = 0.0016).

**Table 3:** Univariate associations of risk factors with first stroke recurrence and stroke recurrence or death.

	Stroke recurrence		Death/stroke recurrence	
	Hazard ratio (95% CI)	*p*	Hazard Ratio (95% CI)	*p*
Age	1.03 (1.01–1.03)	**<0.0001**	1.03 (1.02–1.04)	**<0.0001**
Male	0.87 (0.68–1.11)	0.2671	0.87 (0.68–1.11)	0.2671
History of stroke	3.17 (2.16–4.73)	**<0.0001**	2.14 (1.68–2.73)	**<0.0001**
Stroke subtype				
1 (large-artery atherosclerosis)	ref	ref		
2 (cardioembolism)	0.59 (0.35–0.98)	**0.0477**	0.48 (0.33–0.69)	**0.0001**
3 (small-vessel occlusion)	0.83 (0.32–1.77)	0.6650	1.14 (0.68–1.79)	0.6056
4 (other determined etiology)	0.99 (0.24–2.67)	0.9844	1.00 (0.43–1.99)	0.9952
5 (undetermined etiology)	1.02 (0.57–1.72)	0.9523	1.47 (1.07–2.00)	**0.0164**
NIHSS score arrival	1.02 (0.98–1.06)	0.2594	1.08 (1.060 1.11)	**<0.0001**
tPA	2.07 (1.23–3.31)	**0.0038**	1.51 (1.04 2.12)	**0.0230**
Mechanical thrombectomy	1.49 (0.63–2.97)	0.3100	2.15 (1.38 3.20)	**0.0003**
ICU admission	1.89 (1.28–2.77)	**0.0012**	4.54 (3.55–5.82)	**<0.0001**
Seizures	2.52 (1.63–3.78)	**<0.0001**	1.74 (1.28–2.32)	**0.0003**
Pneumonia	1.58 (1.03–2.37)	**0.0305**	2.79 (2.18–3.55)	**<0.0001**
Brain Edema	1.93 (1.01–3.38)	**0.0311**	3.49 (2.54–4.70)	**<0.0001**
DVT-PE	1.86 (1.01–3.14)	**0.0306**	2.41 (1.72–3.30)	**<0.0001**
Impaired consciousness	0.78 (0.48–1.20)	0.2722	2.38 (1.87–3.03)	**<0.0001**
DM	1.37 (0.88–2.21)	0.1816	1.22 (0.92–1.64)	0.1698
Hypertension	1.95 (1.12–3.73)	**0.0294**	1.50 (1.07–2.16)	**0.0245**
Dyslipidemia	1.70 (1.17–2.50)	**0.0057**	0.84 (0.66–1.07)	0.1599
Atrial fibrillation	1.51 (0.84–2.51)	0.1401	2.18 (1.59–2.94)	**<0.0001**
Dementia before stroke	0.96 (0.37–1.99)	0.9122	1.94 (1.28–2.81)	**0.0009**
Depression	2.56 (1.66–3.85)	**<0.0001**	1.06 (0.73–1.48)	0.7522

CI, confidence interval; NIHSS, National Institutes of Health Stroke Scale; tPA, tissue plasminogen activator; ICU, intensive care unit; DVT-PE, deep vein thrombosis-pulmonary embolism; DM, diabetes mellitus. *p*-values < 0.05 written in bold indicating statistical significant difference.

**Table 4:** Multivariable associations of risk factors with first stroke recurrence and stroke recurrence or death.

	Hazard Ratio (95% CI)	*p*
Model 1	Stroke Recurrence	
History of stroke	3.65 (2.28–5.99)	**0.0001**
tPA	2.84 (1.57–4.86)	**0.0003**
Seizures	1.96 (1.14–3.22)	**0.0105**
Depression	2.26 (1.33–3.69)	**0.0016**
Model 2	Death/Stroke Recurrence	
Age	1.02 (1.01–1.03)	**0.0009**
History of stroke	2.37 (1.74–3.26)	**0.0001**
ICU admission	3.70 (2.63–5.22)	**0.0001**
Pneumonia	1.47 (1.05–2.05)	**0.0249**
Brain Edema	2.36 (1.58–3.46)	**0.0001**

CI, confidence interval; tPA, tissue plasminogen activator; ICU, intensive care unit. *p*-values < 0.05 written in bold indicating statistical significant difference.

### 3.4 Risk factors associated with stroke recurrence/death

Univariate analysis identified several risk factors significantly associated with stroke recurrence. These included age (HR = 1.03, 95% CI: 1.02–1.04, *p* < 0.0001), history of stroke (HR = 2.14, 95% CI: 1.68–2.73, *p* < 0.0001), stroke subtype two vs. one as a reference (HR = 0.48, 95% CI: 0.33–0.69, *p* = 0.0001), stroke subtype five vs. one (HR = 1.47, 95% CI: 1.07–2.00, *p* = 0.0164), initial NIHSS score (HR = 1.08, 95% CI: 1.06–1.11, *p* < 0.0001), tPA treatment (HR = 1.51, 95% CI: 1.04–2.12, *p* = 0.0230), mechanical thrombectomy (HR = 2.15, 95% CI: 1.38–3.20, *p* = 0.0003), ICU admission (HR = 4.54, 95% CI: 3.55–5.82, *p* < 0.0001), seizures (HR = 1.74, 95% CI: 1.28–2.32, *p* = 0.0003), pneumonia (HR = 2.79, 95% CI: 2.18–3.55, *p* < 0.0001), brain edema (HR = 3.49, 95% CI: 2.54–4.70, *p* < 0.0001), DVT-PE (HR = 2.41, 95% CI:1.72–3.30, *p* < 0.0001), impaired consciousness (HR = 2.38, 95% CI:1.87–3.03, *p* < 0.0001) hypertension (HR = 1.50, 95% CI:1.07–2.16, *p* = 0.0245), AF (HR = 2.18, 95% CI: 1.59–2.94, *p* < 0.0001), and dementia (HR = 1.94, 95% CI:1.28–2.81, *p* = 0.0009). Upon multivariable analysis (Table 4), the risk of stroke recurrence/death remained significantly associated with age (HR = 1.02, 95% CI:1.01–1.03, *p* = 0.0009), history of stroke (HR = 2.37, 95% CI:1.74–3.26, *p* = 0.0001), ICU admission (HR = 3.70, 95% CI:2.63–5.22, *p* = 0.0001), pneumonia (HR = 1.47, 95% CI:1.05–2.05, *p* = 0.0249), and brain edema (HR = 2.36, 95% CI:1.58–3.46, *p* = 0.0001).

## 4 Discussion

This study addresses a critical gap in understanding the incidence and risk factors associated with recurrent stroke in Saudi Arabia. The findings revealed that the cumulative risk of recurrent stroke was 2.55% within 30 days, increasing to 5.29% at 90 days, 6.91% at 180 days, and reaching ~10% within 365 days following the index stroke. Our findings align with a prospective cohort study by Modrego et al., reporting a cumulative risk of recurrence of 2.1% at 30 days and 9.5% at 365 days (12). In comparison, a systematic review and meta-analysis reported a pooled cumulative risk of stroke recurrence of 3.1% (95% CI, 1.7–4.4) at 30 days and 11.1% (95% CI, 9.0–13.3) at 365 days (13). Similarly, a meta-analysis by Lin et al. reported a cumulative recurrence risk of 7.7% at 90 days, 9.5% at 180 days, and 10.4% at 365 days. These rates are slightly higher than those observed in our study at 90 and 180 days; however, the 365-day recurrence rate aligns with our findings (14). Variations in study design, inclusion criteria (e.g., different stroke subtypes), patient demographics such as age, and the prevalence of comorbidities may explain the observed discrepancies in recurrence rates. Given the differences in demographics and comorbidities, future research should focus on intracranial atherosclerotic disease (ICAD), as it may represent a significant factor in the etiology that was not explored in this study (15).

Moreover, Rucker et al. and Flach et al. reported a 90-day ischemic stroke recurrence rate of 3.1% (95% CI: 2.5–3.7) and 2.2% (95% CI: 1.8–2.6), respectively, lower than our findings (16, 17). This discrepancy may stem from the inclusion of all stroke types in their studies rather than focusing solely on ischemic stroke. Additionally, differences in ischemic stroke subtype distribution could explain the variation; for instance, subtype one accounted for 50% of strokes in our study, compared to only 8.4% in Rucker et al. and 16% in Flach et al. Furthermore, their studies’ lack of reported comorbidities may contribute to these differences (16, 17). Lastly, Arsava et al., in a multi-center study across the USA, South Korea, and Brazil, reported a 90-day recurrence rate of 4.2% (95% CI: 3.2–5.2), which aligns more closely with our results. Their study also noted that 34% of recurrent strokes were subtype one (18). These findings highlight the significance of considering stroke subtype distribution and comorbidities in interpreting recurrence rate variations across studies.

In the current study, the cumulative incidence rates of death or stroke recurrence were as follows: 8.13% at 30 days, 13.60% at 90 days, 16.69% at 180 days, and 21.83% at 365 days after stroke. Patients with a prior stroke history showed significantly higher incidence rates. These findings align with literature emphasizing elevated risks in individuals with previous stroke history (19). Variations among stroke subtypes also influenced outcomes, consistent with a report linking different subtypes to varying risks of recurrence and mortality. For instance, a previous study indicated that specific ischemic stroke subtypes, such as large artery atherosclerosis and cardioembolic strokes, are associated with higher risks of recurrence compared to other subtypes (20).

Univariate analysis identified multiple predictors of stroke recurrence, including advanced age, history of stroke, tPA treatment, ICU admission, seizures, pneumonia, brain edema, DVT-PE, hypertension, dyslipidemia, and depression. Multivariable analysis confirmed the independent association of stroke recurrence with a history of stroke, tPA treatment, seizures, and depression. Previous history of stroke was identified as an independent predictor of stroke recurrence, consistent with findings by Xu et al., who reported a 2- to 3-fold increased risk of stroke recurrence in patients with prior stroke history (20). This underscores the critical need for tailored secondary prevention in this high-risk population.

The observed association between tPA, an essential treatment for improving acute stroke outcomes, and an increased risk of recurrence highlights the need for further investigation. Potential mechanisms underlying this association may include the influence of underlying stroke etiologies, predisposing factors, or complications related to reperfusion therapies. Future research should focus on elucidating these mechanisms and assessing the long-term impact of tPA on recurrent stroke risk, considering both patient-specific factors and treatment-related variables.

Moreover, post-stroke seizures and depression were independent stroke recurrence predictors, aligning with studies such as Doria et al. and Zeng et al. (21, 22). Seizures likely reflect more significant cortical damage or vascular instability (23), while depression may impact treatment adherence and recovery (24). It is crucial to educate stroke patients with seizures and depression about their risk of recurrence. Similarly, finding ways to control seizures and depression in these patients is vital in clinical settings and future studies.

Similarly, Univariate analysis revealed numerous predictors for stroke recurrence/death, including age, history of stroke, stroke subtypes, initial NIHSS, ICU admission, mechanical thrombectomy, pneumonia, brain edema, and comorbidities like AF and dementia. Multivariable analysis confirmed significant associations with age, history of stroke, ICU admission, pneumonia, and brain edema. Studies have shown that older adults are at increased risk due to age-related vascular changes, comorbidities, and reduced physiological reserve. For example, a meta-analysis by Mohan et al. reported a similar association between advancing age and stroke recurrence rates, underscoring the need for age-tailored secondary prevention strategies (13). Additionally, pneumonia and brain edema, as markers of post-stroke complications, have also been implicated in poorer outcomes. For instance, a study by Finlayson et al. found that post-stroke pneumonia is associated with increased mortality and more extended hospital stays. The study emphasizes the importance of early identification and management of pneumonia in stroke patients to improve outcomes (25). Similarly, a previously published study found that brain edema is an independent predictor of poor outcomes after ischemic stroke (26). Additionally, a review article by Zhang et al. emphasized that cerebral edema is a common complication of acute ischemic stroke, leading to poorer functional outcomes and substantially increasing mortality rates. The review highlights the importance of early identification of patients at high risk for severe edema to implement intensive monitoring and evidence-based interventions (27).

Our study possesses several notable strengths and limitations. A key strength is that, to the best of our knowledge, this is the first study to report the long-term incidence of recurrent stroke and mortality in Saudi Arabia, utilizing a larger sample size. This unique contribution provides a foundation for understanding stroke recurrence and mortality patterns for ischemic stroke patients in this region. Additionally, the study identifies a comprehensive range of risk factors, such as history of stroke, administration of tPA, seizures, and depression, many of which have been underexplored in the existing literature. Another significant strength lies in the study’s inclusion of data from two hospitals located in different regions of Saudi Arabia. This approach enhances the external validity of our findings by capturing a more diverse patient population and reducing the likelihood of regional biases.

However, the study is not without limitations. Its retrospective design inherently relies on the accuracy and completeness of medical record documentation, which may affect the reliability of the results. While we captured recurrence incidence at various time points, data limitations prevented the evaluation of recurrence beyond 1 year, which could have provided a more comprehensive understanding of long-term risks. Moreover, our study did not include data regarding carotid stenosis, surgical interventions (e.g., carotid endarterectomy), or interventional procedures (e.g., stenting). Furthermore, this study did not assess common risk factors, such as smoking and chronic kidney disease, leaving potential gaps in the analysis. Future research should focus on evaluating secondary prevention interventions, patient adherence, and their impact on stroke recurrence to provide a more comprehensive understanding of these critical factors. Further, while our study focused on interventions related to ischemic stroke, we acknowledge that we did not explore the influence of other medication use on stroke recurrence. Future research should consider the comparative effects of treatment versus non-treatment, as well as the potential impact of additional medications, to provide a more comprehensive understanding of factors influencing stroke recurrence. Finally, while our study focused on dyslipidemia and the TOAST criteria of stroke etiology, the lack of data on intracranial atherosclerotic disease (ICAD) is an important gap. We recommend that future studies specifically investigate the role of ICAD in the pathophysiology of stroke among Saudi patients, as it could provide valuable insights. Addressing these omissions will help present a more holistic view of the risk factors contributing to stroke recurrence. Despite these limitations, the study offers valuable insights and establishes a foundation for further investigations in this essential area.

## 5 Conclusion

This study highlights the significant burden of stroke recurrence and mortality among patients within the first year following a stroke, emphasizing the importance of identifying and managing risk factors. Key findings include a stroke recurrence rate of 9.96% and a combined death/stroke recurrence rate of 21.83% within 365 days.

A variety of risk factors were identified, with multivariable analysis revealing that a history of stroke, administration of tPA, seizure, and depression were significantly associated with stroke recurrence. However, only a prior stroke history remained significantly associated with the combined outcome of stroke recurrence and death. Additional risk factors for the composite outcome included older age, ICU admission, pneumonia, and brain edema.

These findings highlight the importance of early, focused interventions to reduce preventable complications and better manage comorbidities. Special attention should be given to patients with a history of stroke or severe presentations, as they are most vulnerable.

Further research is needed to validate these findings and deepen our understanding of the factors contributing to stroke recurrence and related outcomes.

## Data Availability

The data analyzed in this study is available upon request. Requests to access these datasets should be directed to Faisal Alamri, alamrif@ksau-hs.edu.sa.
